# Treatment of Tibial Plateau Fractures with a Circular External Fixator: A Comparative Analysis of Two Assembly Methods

**DOI:** 10.1055/s-0044-1785203

**Published:** 2024-04-10

**Authors:** Leonardo Berto, Gustavo Henrique de Barros Palma, André Crippa da Silva, Mauro Remulo Grinfelder Brunel Rodrigues, Renato Amorim, Gracielle Silva Cardoso

**Affiliations:** 1 Serviço de Ortopedia e Traumatologia, Hospital Governador Celso Ramos, Florianópolis, SC, Brasil; 2 Serviço de Ortopedia e Traumatologia, Posto Médico de Guarnição da 16ª Brigada de Infantaria de Selva, Exército Brasileiro, Tefé, AM, Brasil

**Keywords:** tibial plateau fractures, external fixators, Ilizarov technique, Kirschner wires

## Abstract

**Objective** To compare the functional outcomes of two circular external fixation techniques to treat complex fractures of the proximal end of the tibia.

**Materials and Methods** The present is a retrospective cohort study with 51 patients who underwent surgical treatment for complex fractures of the tibial plateau with a circular external fixator. There were two groups of patients: 12 subjects underwent treatment with the classic assembly technique, and 39 subjects underwent treatment with the simplified technique. The variables analyzed included age, sex, injury mechanism, trauma energy, associated injuries, fixator type, time of fixator use, and clinical-radiographic outcomes. The classic technique mainly uses transfixing Kirschner wires, while the simplified one replaces the Kirschner wires with Schanz pins in the distal block of the circular external fixator.

**Result** There were no statistically significant differences ( *p* > 0.05) between the two groups concerning the clinical-radiographic outcomes, including fracture consolidation, quality of joint fracture reduction, range of motion, lower limbs residual discrepancy, and postoperative pain.

**Conclusion** We suggest that the simplified technique, using Schanz pins instead of Kirschner wires, can be a viable and effective alternative to treat complex fractures of the proximal end of the tibia with a circular external fixator. This simplified approach can offer benefits, such as a lower infection rate and greater patient comfort, without compromising clinical and radiographic outcomes, thus justifying its use.

## Introduction

Fracture of the proximal end of the tibia historically represents a spectrum of injuries with complex treatment. In bicondylar injuries and those with metaphyseal-diaphyseal dissociation, complications inherent to traditional treatment methods are invariably significant. Therefore, research focuses on alternative forms with less invasive approaches to preserve the biological and anatomical structures of the affected region to reduce the chances of complications. Currently, with the advent of minimally-invasive techniques, it is believed that the reestablishment of axial alignment of the lower limb is more important than the absolute restoration of joint congruity in the therapeutic outcome of tibial plateau fractures. 1 2 3

Ilizarov developed the circular external fixation technique to treat difficult-to-manage orthopedic issues. The method is based on the biological principles of bone consolidation and a circular external fixator with rings and transfixing tensioned Kirschner wires for bone fixation. 4

Efforts have been made to simplify device application and configuration to improve patient comfort while maintaining the appropriate combination of system stability and dynamics. Reducing the number of wires in the assembly reduces the infection rate, improves patient comfort during treatment, and, at the same time, reduces device stability. Introducing Schanz pins to Ilizarov circular external fixator assemblies enables a more simplified configuration of a sufficiently-rigid apparatus and reduces wire-related soft tissue complications. 5

Cardoso et al. 6 performed a biomechanical comparison of two circular external fixator assemblies in composite bone models to treat these fractures: the classic assembly (mainly using Kirschner wires) and a proposed simplified assembly which mainly uses Schanz pins. These authors observed that both assemblies had similar biomechanical behaviors.

Therefore, the present study aimed to compare the functional outcomes in a series of cases using the classic assembly or the proposed assembly with more Schanz pins to evaluate the feasibility of a simplified circular external fixator to treat tibial proximal end fractures.

We hypothesized that the simplified assembly would not alter the clinical-radiographic outcomes compared with the classic assembly. As such, justifying the use of the simplified assembly to treat complex fractures of the tibial plateau.

## Materials and Methods

The present retrospective, quantitative, and comparative study used medical records from patients diagnosed with tibial plateau fractures classified as Schatzker V and VI 7 who underwent surgical treatment with a circular external fixator.

The institutional Ethics in Research Committee approved the study under number CAAE: 52077521.7.0000.5360.

### Cohort and Sample

The sample consisted of all patients (51) with fractures of the tibial plateau classified as Schatzker V and VI 7 from March 2012 to June 2021 who underwent surgical treatment with a circular external fixator. The patient's medical records supplied the extracted data.

**Fig. 1:**
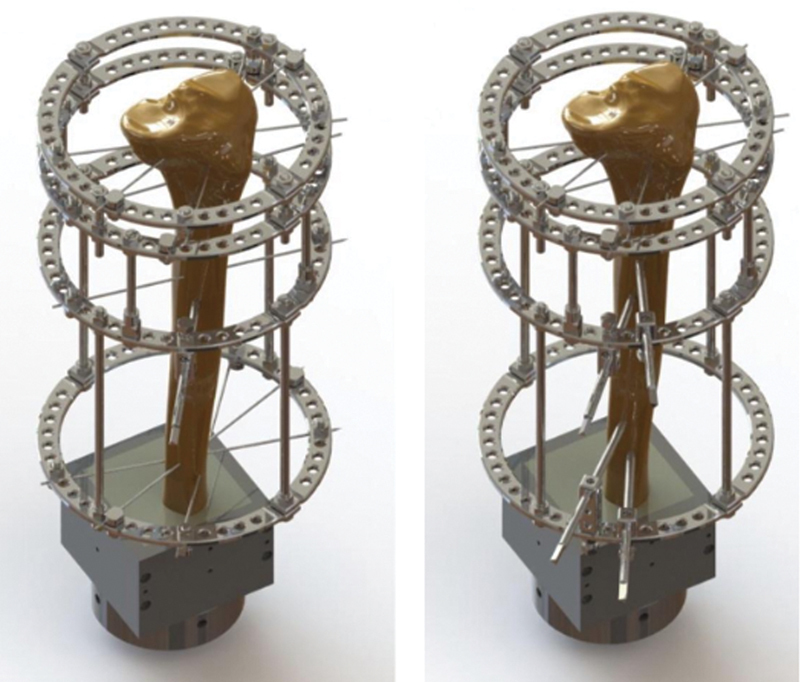
Illustration of the classic (left) and simplified (right) assemblies demonstrating the positioning of the Kirschner wires and Schanz pins.

The outcomes of patients treated with the classic Ilizarov circular external fixator, which mainly uses transfixing Kirschner wires (n = 12), were compared with those of patients treated using a simplified assembly, which replaces the Kirschner wires of the distal block by Schanz pins (n = 39). Fig. 1 illustrates both assemblies.

The descriptive variables evaluated were age, sex, injury mechanism, trauma energy, associated injuries, fixator type, time of fixator use, and postoperative follow-up time.

The inferential analyses included the following: external fixator usage time in months, consolidation and presence of infection, quality of joint fracture reduction, range of motion, residual limb discrepancy and postoperative pain at the end of the follow-up.

Patients who lost to follow-up or treated with a transarticular circular external fixator were excluded. Patients still using circular external at the time of data collection fixator, regardless of the assembly, were also excluded.

A group of three orthopedic surgeons, two of whom members of the Association for the Study and Application of the Method of Ilizarov (ASAMI) and the Brazilian Society of Orthopedics and Traumatology (SBOT, for its acronym in Portuguese [ *Sociedade Brasileira de Ortopedia e Traumatologia* ]), and one SBOT member, performed the radiological analysis of joint deviation. The radiological results were divided into good alignment, misalignment up to 2 mm, misalignment ranging from 2 mm to 4 mm, and misalignment higher than 4 mm.

### Statistical Analysis

After defining the variables and collecting data, results were pooled in a Microsoft Office Excel spreadsheet (Microsoft Corp., Redmond, WA, United States), version 12.0 and analyzed using the Jamovi software (open source), version 1.2. First, descriptive analyses determined means and confidence intervals for numerical variables and absolute and relative frequency measures for categorical variables. Next, we proceeded to the inferential analyses.

The Mann-Whitney U test verified differences between fixator use time and the postoperative follow-up time in both groups given the non-normality of the dependent variable and the sample size. The remaining analyses used the Chi-squared association test because the variables were categorical. The Fisher exact test analyzed selected associations with cells with absolute values lower than 5, and the continuity correction (Yates) test determined associations in cells with an absolute value of 0. For all hypothesis tests, the significance level was of 5%.

## Results

We analyzed the medical records of a total of 82 patients. The exclusion criteria were transarticular assembly (n = 6) and loss at follow-up (n = 25); therefore, 51 patients remained in the sample.

**Fig. 2:**
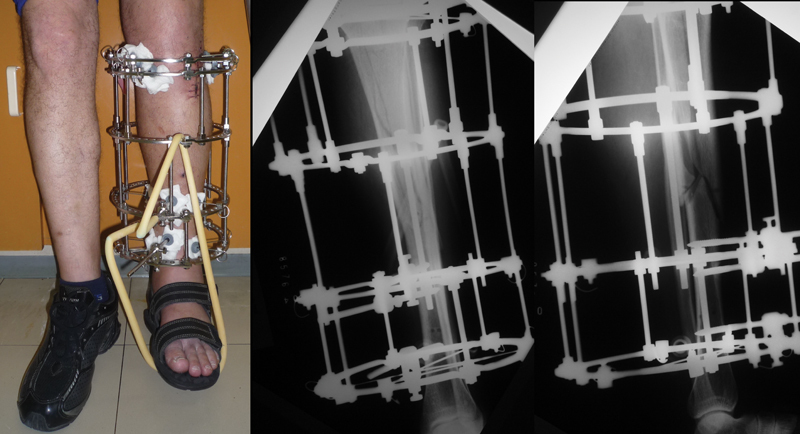
Clinical and radiographic images demonstrating the classic assembly in a patient included in the study.

**Fig. 3:**
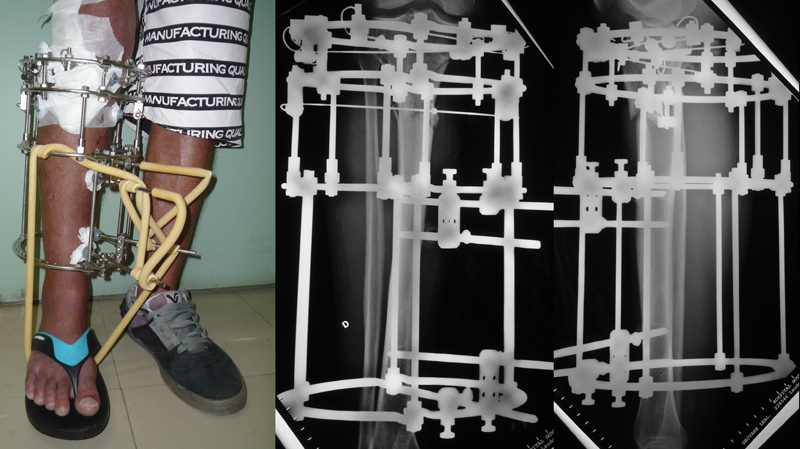
Clinical and radiographic images demonstrating the simplified assembly in a patient included in the study.

Regarding fixators, 12 patients (23.5%) underwent the classic assembly ( Fig. 2 ), and 39 (76.5%), the simplified assembly ( Fig. 3 ). The analysis compared the outcomes of these two groups of patients.

The mean age of the patients treated with the classic and simplified assemblies was of 46.3 and 45.8 years respectively, with no statistically significant difference between the groups. Both groups had a predominance of male subjects, with no statistically significant difference in sex distribution.

In both groups, there was a predominance of high-energy trauma, mostly traffic accidents, with no statistically significant difference between them. Of the total number of associated injuries, most proximal tibial fractures were isolated (52.3%), followed by associated fractures of the femur (proximal, diaphyseal, or neck fractures) in 8.4% of the patients.

**Fig. 4:**
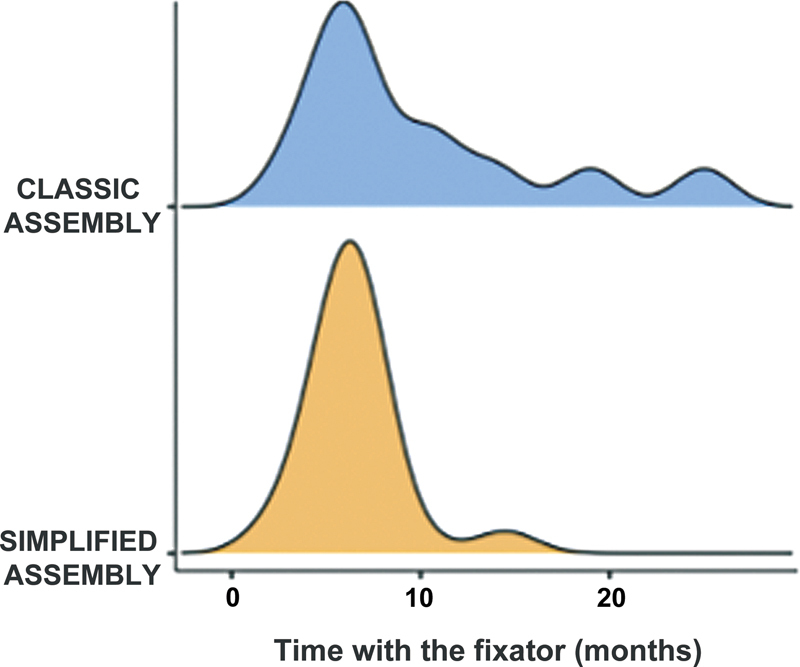
Graph showing the temporal distribution of the use of the external fixator in the classic and simplified assembly groups.

The average time of fixator use, regardless of the assembly, was of 7.25 months. The type of fixator assembly, whether classic or simplified, did not determine a statistically significant difference in the time of use ( *p* = 0.171) per the temporal distribution shown in Fig. 4 .

The average postoperative follow-up was of 48.5 months in the classic group, and of 42.7 months in the simplified group, with no statistically significant difference ( *p* = 0.629).

Two variables determined the quality of the treatment outcome: union without infection, and non-union and/or infection. Consolidation with no infection occurred in 83.3% of the patients in the classic group, and in 94.9% of the subjects in the simplified group. Table 1 shows the lack of statistically significant difference ( *p* = 0.232) regarding the Fisher exact test between both groups.

**Table 1:** Fisher exact test comparing the therapeutic outcome in patients submitted to the classic and simplified assemblies

	Outcome		
Fixator type	Non-union or infection	Consolidation with no infection	Total
Classic	2	10	12
	16.7%	83.3%	100%
Simplified	2	37	39
	5.1%	94.9%	100%
Total	4	47	51
	7.8%	92.2%	100%

Note: Fisher exact test ( *p* = 0.232).

In the classic group, 66.7% of the patients had good radiographic outcomes (that is, proper alignment or misalignment of up to 2 mm), while 71.8% of those in the simplified group presented good radiographic outcomes, with no statistically significant difference ( *p* = 0.65).

Neither did the range of motion of the knee joint after fixator removal show statistical differences between the different assemblies ( *p* = 0.826), as shown in Table 2 , considering that the range of motion was higher than 80° among 83.3% of the classic group and in 87.8% of those in the simplified group.

**Table 2:** Fisher exact test comparing the range of motion of the knee joint after external fixator removal in patients treated with the classic and simplified assemblies

	Knee range of motion				
Fixator type	< 80°	80°–109°	110°–130°	> 130°	Total
Classic	2	5	3	2	12
	16.7%	41.7%	25%	16.7%	100%
Simplified	5	19	11	4	39
	12.8%	48.7%	28.2%	10.3%	100%
Total	7	24	14	6	51
	13.7%	47.1%	27.5%	11.8%	100%

Note: Fisher exact test ( *p* = 0.826).

Regarding the residual discrepancy between limbs, there was no statistical significance ( *p* = 0.51 in the Fisher exact test associated with the Yates continuity correction test), as 83.3% of the patients in the classic group and 92.3% of the simplified group had no dysmetria between the limbs.

When evaluating residual pain after external fixator removal, there was no statistically significant difference ( *p* = 0.893) between the groups, as shown in Table 3 , considering that 25% of the classic group and 30.6% of the simplified group complained of moderate or severe postoperative pain.

**Table 3:** Fisher exact test comparing pain after external fixator removal in patients treated with the classic and simplified assemblies

	Pain				
Fixator type	Absent	Mild	Moderate	Severe	Total
Classic	8	1	3	0	12
	66.7%	8.3%	25%	0%	100%
Simplified	20	7	10	2	39
	51.3%	17.9%	25.6%	5.1%	100%
Total	28	8	13	2	51
	54.9%	15.7%	25.5%	3.9%	100%

Note: Fisher exact test ( *p* = 0.893).

## Discussion

Based on clinical experience and biomechanical studies, for severe tibial plateau fractures, the best stabilization of each bone fragment using circular external fixation occurs with two-level fixation and four wires inserted at right angles. However, in most clinical situations, it is not possible to position the wires at right angles due to anatomical limitations. Reducing the number of wires or the angle between them affects the stability of the bone fragment fixation, which ultimately can hinder the success of the orthopedic treatment. 8

The literature is not consensual regarding the type of external fixation to treat these fractures. There are descriptions of the use of circular fixators and exclusively Kirschner wires, circular fixators and various associations of Kirschner wires and Schanz pins, and fixators that use bars and rings with Kirschner wires in the metaphysis and exclusively Schanz pins in the diaphysis. 9

Reducing the number of wires in the assembly reduces the infection rate and improves patient comfort during treatment, simultaneously reducing device stability. The introduction of Schanz pins to Ilizarov circular external fixator assemblies enables the configuration of a more rigid device and a reduction in wire-associated soft tissue complications. 5 As such, this simplified assembly was proposed to replace the Kirschner wires from the distal block with two Schanz pins for each ring, as shown in Fig. 1 .

In the present study, the average age of the patients in the classic and simplified groups was of 46.3 and 45.8 years respectively, and most subjects were male. The average time of fixator use was of 7.25 months. Ghimire et al. 10 found similar data in their study, with a predominance of male patients and an average age of 39.98 years, but with an estimated time until fixator removal of 15.09 weeks. In both studies, traffic accidents were the main cause of fractures.

Ali et al. 9 studied a standardized assembly for complex tibial plateau fractures and observed an average time of fixator use of 18 weeks. In our study, 23.5% of the patients used the classic fixator, and 76.5% used the simplified device, with no difference in postoperative range of motion between the groups. The range of motion at the end of the treatment with both assemblies was higher than 80° in more than 80% of the cases. Ali et al. 9 reported an average range of motion at the end of treatment of 112°.

In the present study, most proximal tibial fractures were isolated (52.3%), followed by associated (proximal, diaphyseal, or neck) femoral fractures. The radiological analysis of fracture consolidation, the quality of joint surface reduction, and the residual discrepancy between limbs presented no statistically significant differences between the groups.

We compared our findings with those of a 2006 multicenter, prospective, randomized study from the Canadian Orthopaedic Trauma Society. 11 Both studies evaluated different treatment approaches for fractures. We focused on comparing two types of external fixator assembly for proximal tibial fractures, while the Canadian Orthopaedic Trauma Society compared treatment with a circular fixator versus open reduction and internal fixation in patients with several fractures. These studies suggest that different external fixator assemblies and a circular fixator compared to open reduction and internal fixation can be viable options to treat fractures. However, it is necessary to consider patient characteristics, the nature of the fracture, and individual clinical features when deciding the most appropriate treatment approach. Therefore, analyzing the two studies, the simplified circular external fixator appears to be a good alternative to treat proximal tibial fractures, resulting in outcomes consistent with those of other studies 11 concerning the quality of the treatment outcome and associated complications.

The main complications related to high-energy tibial plateau fractures are compartment syndrome, neurovascular injury, and skin necrosis. The postoperative infection rate with open reduction can range from 5% to 20%. 12 13 The percutaneous assembly reduces the risk of periosteal injury and preserves local biology. Both groups presented similar complications concerning loss of joint mobility, postoperative residual pain, and limb length asymmetry. Regarding the therapeutic outcome, there was no significant difference between the two types of assembly and the literature in terms of non-union, infection, or both. 11 14

The present study shows that an assembly using Schanz pins results in a high consolidation rate and low risk for deep infection, which is consistent with the literature. 15 16

Due to its retrospective nature, the present study has critical limitations, including the disparity between the cohorts studied, with a predominance of simplified assemblies, and the limited number of patients and high rate of loss to follow-up. Therefore, further studies are required to minimize selection bias.

## Conclusion

Since there is no current consensus to define the ideal type of assembly of the circular external fixator to treat severe tibial plateau fractures and the increased focus on finding less invasive and biologically-aggressive treatments, the simplified device is a viable and promising option.

Given that there were no statistical differences between the compared groups, we suggest the simplified technique for the cases herein described. In addition, new studies may provide more robust evidence for this technique.
