# Supplementary material for: Treatment of Tibial Plateau Fractures with a Circular External Fixator: A Comparative Analysis of Two Assembly Methods
Source: Rev Bras Ortop (Sao Paulo). 2024 Apr 10;59(2):e206–12. doi: 10.1055/s-0044-1785203 (PMC11006512; doi:10.1055/s-0044-1785203)
Supplement: Related texts

# Research article: Tratamento de fraturas do planalto tibial com fixador externo circular: Uma análise comparativa de duas formas de montagem

**Keywords:** fraturas do planalto tibial, fixadores externos, técnica de Ilizarov, fios de Kirschner

## Abstract

**Objetivo** Comparar os resultados funcionais entre duas técnicas de fixação externa circular utilizadas no tratamento de fraturas complexas da extremidade proximal da tíbia.

**Materiais e Métodos** Trata-se de um estudo de coorte retrospectivo, com 51 pacientes submetidos ao tratamento cirúrgico de fraturas complexas do planalto tibial com fixador externo circular. Os pacientes foram divididos em dois grupos: 12 pacientes tratados com a técnica clássica e 39 pacientes tratados com a técnica simplificada. As variáveis analisadas incluíram idade, sexo, mecanismo da lesão, energia do trauma, lesões associadas, tipo de fixador, tempo de uso do fixador e resultados clínico-radiográficos. A técnica clássica lança mão principalmente de fios de Kirschner transfixantes, e a simplificada substitui os fios de Kirschner por pinos de Schanz no bloco distal do fixador externo circular.

**Resultados** A partir da comparação das montagens, não encontramos diferenças estatisticamente significativas ( *p* > 00,5) entre os dois grupos em relação aos resultados clínico-radiográficos, incluindo a consolidação da fratura, a qualidade da redução da fratura articular, a amplitude de movimento, a discrepância residual na medida dos membros inferiores e a dor do paciente no pós-operatório.

**Conclusão** Sugerimos que a técnica simplificada, utilizando pinos de Schanz no lugar dos fios de Kirschner, pode ser uma alternativa viável e eficaz no tratamento de fraturas complexas da extremidade proximal da tíbia com fixador externo circular. Essa abordagem simplificada pode oferecer benefícios, como menor taxa de infecção e maior conforto para o paciente, sem comprometer os resultados clínicos e radiográficos, o que justifica, o seu uso.

## Introdução

A fratura da extremidade proximal da tíbia historicamente representa um espectro de lesões com alta dificuldade de tratamento. Quando se avaliam as lesões bicondilares e com dissociação metáfiso-diafisária, sabe-se que as complicações inerentes aos métodos tradicionais de tratamento são invariavelmente importantes. Portanto, tem-se procurado formas alternativas com abordagens menos invasivas a fim de preservar as estruturas biológica e anatômica da região acometida para diminuir, assim, as chances de complicações. Atualmente, com o advento de técnicas minimamente invasivas, acredita-se que o restabelecimento do alinhamento axial do membro inferior seja mais importante do que a restauração absoluta da congruência articular no resultado do tratamento das fraturas do planalto tibial. 1 2 3

Ilizarov desenvolveu a técnica de fixação externa circular para o tratamento de problemas ortopédicos de difícil manejo. O método se baseia nos princípios biológicos da consolidação óssea e no uso de um fixador externo de formato circular que utiliza anéis e fios de Kirschner tensionados transfixantes para a fixação óssea. 4

Esforços têm sido feitos para simplificar a aplicação e a configuração dos aparelhos para melhorar o conforto dos pacientes enquanto se mantém a apropriada combinação de estabilidade e dinâmica do sistema. A redução do número de fios na montagem diminui a taxa de infecção e melhora o conforto do paciente durante o tratamento, mas simultaneamente diminui a estabilidade do aparelho. A introdução de pinos de Schanz às montagens de fixadores externos circulares de Ilizarov propicia a configuração mais simplificada de um aparelho suficientemente rígido e uma redução das complicações de partes moles associadas aos fios. 5

Cardoso et al. 6 compararam biomecanicamente dois tipos de montagem de fixador externo circular em modelos de ossos compósitos para o tratamento dessas fraturas: uma montagem considerada clássica (que emprega principalmente fios de Kirschner) e uma montagem proposta, considerada simplificada (que utiliza majoritariamente pinos de Schanz), e observaram que ambas apresentaram comportamentos biomecânicos semelhantes.

Portanto, este trabalho tem por objetivo comparar os resultados funcionais de uma série de casos em que se utilizou a montagem clássica ou a proposta com mais pinos de Schanz, a fim de avaliar a possibilidade de utilização de uma técnica mais simplificada do fixador externo circular para o tratamento dessas fraturas.

Dessa forma, considera-se a hipótese de que a montagem simplificada, quando comparada com a clássica, não alteraria os resultados clínico-radiográficos, o que justificaria o uso dessa montagem para o tratamento de fraturas complexas do planalto tibial com fixador externo circular.

## Materiais e Métodos

Este é um estudo retrospectivo, quantitativo, comparativo, que usou arquivos de prontuários médicos dos pacientes com diagnóstico de fraturas do planalto tibial classificadas como Schatzker V e VI, 7 que foram submetidos a tratamento cirúrgico com fixador externo circular.

O estudo foi aprovado pelo pelo Comitê de Ética e Pesquisa de nossa instituição sob o número CAAE: 52077521.7.0000.5360.

## Coorte e Amostra

A amostra deste estudo foi composta por todos os pacientes (51) que sofreram fraturas do planalto tibial classificadas como Schatzker V e VI 7 de março de 2012 a junho de 2021, submetidos a tratamento cirúrgico com fixador externo circular. Os dados foram acessados pelo arquivo dos prontuários dos pacientes.

Foram comparados os desfechos dos pacientes submetidos à montagem clássica do fixador externo circular de Ilizarov, que utiliza majoritariamente fios de Kirschner transfixantes (n = 12), com os dos pacientes submetidos a uma montagem simplificada, que substitui os fios de Kirschner do bloco distal por pinos de Schanz (n = 39). A Fig. 1 ilustra ambas as montagens utilizadas.

**Fig. 1:** Ilustração das montagens clássica (à esquerda) e simplificada (à direita) demonstrando o posicionamento dos fios de Kirschner e dos pinos de Schanz utilizados.

As variáveis descritivas avaliadas foram idade, sexo, mecanismo da lesão, energia do trauma, lesões associadas, tipo do fixador, tempo de uso do fixador e tempo de seguimento pós-operatório.

Quanto às análises inferenciais, foram avaliados: o tempo de uso em meses, a presença de infecção, a qualidade da redução da fratura articular, a amplitude de movimento, a discrepência residual na medida dos membros inferiores e a dor ao final do seguimento pós-operatório.

Foram excluídos pacientes com perda de seguimento ou pacientes tratados com fixador externo circular transarticular. Também foram excluídos pacientes que ainda estavam em uso do fixador externo circular, seja qual fosse sua montagem.

A análise radiológica do desvio articular foi feita por um grupo de três ortopedistas, sendo dois com área de atuação em reconstrução e alongamento ósseo, membros da Associação para o Estudo e Aplicação dos Métodos de Ilizarov (ASAMI) e da Sociedade Brasileira de Ortopedia e Traumatologia (SBOT), e um membro da SBOT. Os resultados radiológicos foram divididos em bom alinhamento, desalinhamento de até 2 mm, desalinhamento entre 2 mm e 4 mm e desalinhamento maior do que 4 mm.

## Análise Estatística

Após a definição das variáveis e a coleta de dados, sua computação foi realizada por meio de planilha do Microsoft Office Excel (Microsoft Corp., Redmond, WA, Estados Unidos) versão 12.0, e as análises, a partir do programa Jamovi (código aberto), versão 1.2. Inicialmente, foram feitas análises descritivas com o uso de médias e intervalos de confiança para as variáveis numéricas e medidas de frequência absoluta e relativa para variáveis categóricas. Em seguida, foram feitas as análises inferenciais.

Para verificar diferenças entre o tempo transcorrido com utilização do fixador e o tempo de seguimento pós-operatório nos dois grupos, optou-se pelo teste U de Mann-Whitney, dada a não normalidade da variável dependente e o tamanho da amostra. Nas demais análises, optou-se pelo teste de associação de qui-quadrado, uma vez que as demais variáveis eram categóricas. Para determinadas associações, cujas caselas apresentaram valores absolutos inferiores a 5, optou-se pelo teste exato de Fischer; já para associações com caselas de valor absoluto 0, foi aplicada a correção de continuidade (Yates). Em todos os testes de hipóteses, o nível de significância considerado foi de 5%.

## Resultados

Foram coletadas informações de prontuários médicos de um total de 82 pacientes. Os critérios de exclusão foram montagem transarticular (n = 6) e perda de seguimento (n = 25), de modo que restaram 51 pacientes na amostra.

Quanto à montagem dos fixadores, a técnica clássica foi utilizada em 12 pacientes (23,5%), conforme ilustrado na Fig. 2 , e a técnica com pino de Schanz, em 39 (76,5%), como demonstrado na Fig. 3 . A análise foi uma comparação dos resultados obtidos por esses dois grupos de pacientes.

**Fig. 2:** Imagem clínica e radiográfica demonstrando a montagem clássica em paciente incluído no estudo.

**Fig. 3:** Imagem clínica e radiográfica demonstrando a montagem simplificada em paciente incluído no estudo.

A média de idade dos pacientes tratados com a montagem clássica foi de 46,3 anos, e a dos pacientes tratados com a montagem simplificada foi de 45,8 anos, não havendo diferença estatisticamente significativa entre os grupos. Houve predominância do sexo masculino em ambos os grupos, não havendo diferença estatística significativa na distribuição de sexo entre os grupos.

Em ambos os grupos houve predominância de traumas de alta energia, majoritariamente acidentes de trânsito, não havendo diferença estatisticamente significativa entre os grupos. Do total de lesões associadas avaliadas, a maior parte das fraturas da tíbia proximal foram isoladas (52,3%), seguidas de fratura associada do fêmur (proximal, diafisária ou do colo), em um total de 8,4% dos pacientes.

A média de tempo de uso do aparelho, independentemente da montagem, foi de 7,25 meses. O tipo de montagem não determinou diferença estatisticamente significativa no tempo de uso do aparelho ( *p* = 0,171), conforme distribuição temporal demonstrada na Fig. 4 .

**Fig. 4:** Gráfico demonstrando a distribuição de tempo de uso do fixador externo entre os grupos clássico e simplificado.

A média de seguimento pós-operatório no grupo submetido à montagem clássica foi de 48,5 meses, ao passo que no grupo tratado com a montagem simplificada foi de 42,7 meses; essa diferença não se mostrou estatisticamente significativa ( *p* = 0,629).

Quanto à qualidade do resultado do tratamento, foram usadas duas variáveis: união sem infecção, e não união e/ou infecção. Entre os pacientes submetidos à montagem clássica, observou-se que 83,3% evoluíram com consolidação sem infecção, ao passo que esse número foi de 94,9% grupo da montagem simplificada. Não houve diferença estatisticamente significativa ( *p* = 0,232) no teste exato de Fischer entre os grupos, conforme demonstrado na Tabela 1 .

**Tabela 1:** Teste exato de Fischer comparando o resultado do tratamento entre os grupos clássico e simplificado pino de Schanz

	Resultado		
Tipo de fixador	Não consolidação ou infecção	Consolidação sem infecção	Total
Clássico	2	10	12
	16,7%	83,3%	100%
Simplificado	2	37	39
	5,1%	94,9%	100%
Total	4	47	51
	7,8%	92,2%	100%

Nota: Teste exato de Fisher ( *p* = 0,232).

No grupo da montagem clássica, observou-se que 66,7% apresentavam bom resultado radiográfico (considerado como bom alinhamento ou desalinhamento de até 2 mm), ao passo que esse número foi de 71,8% no grupo da montagem simplificada, sem diferença estatisticamente significativa ( *p* = 0,65).

Os resultados referentes à amplitude de movimento da articulação do joelho após a retirada do fixador tampouco apresentaram diferenças estatísticas entre as diferentes montagens ( *p* = 0,826), conforme demonstrado na Tabela 2 , considerando que no grupo da montagem clássica 83,3% dos pacientes apresentavam amplitude de movimento maior do que 80°, em comparação com 87,8% dos pacientes da montagem simplificada.

**Tabela 2:** Teste exato de Fischer comparando a amplitude de movimento da articulação do joelho após a retirada do fixador externo entre os grupos clássico e simplificado

	Amplitude de movimento de joelho				
Tipo de fixador	< 80°	80°–109°	110°–130°	> 130°	Total
Clássico	2	5	3	2	12
	16,7%	41,7%	25%	16,7%	100%
Simplificado	5	19	11	4	39
	12,8%	48,7%	28,2%	10,3%	100%
Total	7	24	14	6	51
	13,7%	47,1%	27,5%	11,8%	100%

Nota: Teste exato de Fisher ( *p* = 0,826).

Com relação à discrepância residual entre membros, comparando os resultados entre os grupos, não houve significância estatística ( *p* = 0,51 no teste exato de Fischer associado à correção de continuidade de Yates), sendo que em 83,3% dos pacientes da montagem clássica e em 92,3% dos da montagem simplificada não foi observada dismetria entre os membros.

Na avaliação da dor residual após a retirada do fixador externo, também não houve diferença estatisticamente significativa ( *p* = 0,893) entre os grupos, conforme demonstrado na Tabela 3 , considerando que 25% dos pacientes da montagem clássica apresentavam dor moderada ou severa no pós-operatório, em comparação com 30,6% dos pacientes da montagem simplificada.

**Tabela 3:** Teste exato de Fischer comparando a dor após a retirada do fixador externo entre os grupos clássico e simplificado

	Dor				
Tipo de fixador	Ausente	Leve	Moderada	Severa	Total
Clássico	8	1	3	0	12
	66,7%	8,3%	25%	0%	100%
Simplificado	20	7	10	2	39
	51,3%	17,9%	25,6%	5,1%	100%
Total	28	8	13	2	51
	54,9%	15,7%	25,5%	3,9%	100%

Nota: Teste exato de Fisher ( *p* = 0,893).

## Discussão

Com base na experiência clínica e em estudos biomecânicos, no caso das fraturas graves do planalto tibial, a melhor estabilização de cada fragmento ósseo por meio da fixação externa circular é obtida com a fixação em dois níveis e quatro fios inseridos em ângulos retos. Entretanto, na maioria das situações clínicas, não é possível posicionar os fios em ângulos retos devido a limitações anatômicas. A redução do número de fios ou do ângulo entre os fios afeta a estabilidade da fixação dos fragmentos ósseos, o que em última instância pode afetar o sucesso do tratamento ortopédico. 8

Não existe consenso na literatura a respeito do tipo de fixação externa a ser utilizada no tratamento dessas fraturas. Há descrição de utilização de fixadores circulares e exclusivamente fios de Kirschner, fixadores circulares e variadas associações de fios de Kirschner e pinos de Schanz, e fixadores que utilizam barras e anéis com fios de Kirschner na metáfise e exclusivamente pinos de Schanz na diáfise. 9

A redução do número de fios na montagem diminui a taxa de infecção e melhora o conforto do paciente durante o tratamento, mas simultaneamente diminui a estabilidade do aparelho. A introdução de pinos de Schanz às montagens de fixadores externos circulares de Ilizarov permite a configuração de um aparelho mais rígido e uma redução das complicações de partes moles associadas aos fios. 5 Com base nisso, foi proposta uma montagem simplificada, em que os fios de Kirschner do bloco distal são substituídos por dois pinos de Schanz para cada um dos anéis, conforme ilustrado da Fig. 1 .

Neste estudo, a média de idade dos pacientes com as montagens clássica e simplificada foi de 46,3 e 45,8 anos, respectivamente, com predominância do sexo masculino. O tempo médio de uso do aparelho foi de 7,25 meses. Ghimire et al. 10 encontraram dados semelhantes, com predominância do sexo masculino e média de idade de 39,98 anos, mas com tempo estimado até a remoção dos fixadores de 15,09 semanas. Em ambos os estudos os acidentes de trânsito foram a causa principal das fraturas.

Ali et al. 9 realizaram estudo com uma montagem padronizada para fraturas complexas do planalto tibial, e observaram tempo médio de uso do aparelho de 18 semanas. Em nosso estudo, a montagem, clássica foi utilizada em 23,5% dos pacientes, ao passo que a simplificada, em 76,5%, sem diferença entre os grupos quanto à amplitude de movimento pós-operatória. A amplitude de movimento ao final do tratamento em ambas as montagens foi superior a 80° em mais de 80% dos casos, ao passo que no estudo estudo de Ali et al. 9 a amplitude de movimento média ao final do tratamento foi de 112°.

Neste estudo, a maioria das fraturas da tíbia proximal foi isolada (52,3%), seguida de fraturas associadas do fêmur (proximal, diafisária ou do colo). A análise radiológica da consolidação da fratura, a qualidade da redução da superfície articular, e a discrepância residual entre membros não apresentaram diferenças estatisticamente significativas entre os grupos.

Em comparação do nosso trabalho com um estudo multicêntrico, prospectivo e randomizado publicado em 2006 pela Canadian Orthopaedic Trauma Society, 11 os dois estudos avaliaram diferentes abordagens de tratamento para fraturas, sendo o nosso centrado na comparação entre dois tipos de montagem do fixador externo para fraturas da tíbia proximal, e o segundo, na comparação do uso de fixador circular com a redução aberta e fixação interna em pacientes com fraturas variadas. Os estudos sugerem que tanto o uso de diferentes montagens do fixador externo quanto a utilização do fixador circular em comparação à redução aberta e fixação interna podem ser considerados opções viáveis para o tratamento de fraturas. No entanto, é necessário levar em conta as características do paciente, a natureza da fratura e considerações clínicas individuais ao decidir a abordagem de tratamento mais adequada. Dessa forma, analisando os dois estudos, o fixador externo circular simplificado mostra-se uma boa alternativa para o tratamento de fraturas da tíbia proximal, pois mostrou resultados semelhantes aos dos demais estudos 11 em relação à qualidade do resultado do tratamento e às complicações associadas.

As principais complicações relacionadas a fraturas do planalto tibial de alta energia são síndrome compartimental, lesão neurovascular e necrose da pele. A taxa de infecção pós-operatório com a redução aberta pode girar em torno de 5% a 20%. 12 13 Com a montagem percutânea, diminui-se o risco de lesão ao periósteo e mantém-se preservada a biologia local. Em ambos os grupos estudados, foram encontradas complicações semelhantes no que diz respeito à perda da mobilidade articular, dor residual pós-operatória e assimetria do comprimento dos membros. Em relação ao resultado do tratamento, não houve diferença significativa entre os dois tipos de montagem e o encontrado na literatura em termos de não-união e/ou infecção. 11 14

Neste trabalho, evidenciou-se que a montagem com a utilização de pinos de Schanz é uma técnica com alta taxa de consolidação e com baixo risco para infecção profunda, de acordo com o que é encontrado na literatura. 15 16

Por se tratar de um estudo retrospectivo, uma limitação importante foi a disparidade entre as coortes estudadas, com predomínio das montagens simplificadas. Além disso, a natureza do estudo determinou um número limitado de pacientes, com perda elevada de seguimento. Desse modo, deve-se considerar a realização de novos estudos a fim de minimizar o viés de seleção.

## Conclusão

Visto não haver consenso atualmente quanto ao tipo ideal de montagem do fixador externo circular no tratamento de fraturas graves do planalto tibial, e, associado a isso, o objetivo de buscar cada vez mais tratamentos menos invasivos e agressivos biologicamente, a montagem simplificada se torna uma opção viável e promissora.

Tendo em vista não haver diferenças estatísticas de resultados entre os grupos comparados, sugerimos a técnica simplificada nos casos descritos neste trabalho. Além disso, novos estudos podem trazer evidências mais robustas à esta técnica.
